# In-hospital and post-discharge mortality among infants with respiratory syncytial virus in rural Kenya: a 25-year retrospective cohort study

**DOI:** 10.1016/j.langlo.2026.104003

**Published:** 2026-07-10

**Authors:** Maureen W Mburu, Esther Nyadzua Katama, Mercy S Safari, Timothy O Makori, Elijah T Gicheru, Martin Mutunga, Clement Lewa, Robinson Cheruiyot, Hillary Wafula, Grace Maina, Caroline Ngetsa, Augustus Kea, Neema Mturi, Mohammed Shebe, John Kalama Fondo, Charles Chesaro, Hiza Dayo, Joyce U Nyiro, Charles N Agoti, Charles J Sande

**Affiliations:** a Bioscience Department, Kenya Medical Research Institute (KEMRI)–Wellcome Trust Research Programme, Kilifi, Kenya; b Epidemiology and Demography Department, Kenya Medical Research Institute (KEMRI)–Wellcome Trust Research Programme, Kilifi, Kenya; c Kenya Medical Research Institute, Centre for Geographic Medicine Research, Kilifi, Kenya; d Kilifi County Referral Hospital, Kilifi, Kenya; e Centre for Tropical Medicine and Global Health, University of Oxford, Oxford, UK

## Abstract

**Background:**

Respiratory syncytial virus (RSV) is the leading cause of hospital admission for lower respiratory infection in infants worldwide, with more than 95% of deaths occurring in low-income and middle-income countries. Predictors of adverse outcomes following RSV hospitalisation remain poorly defined. We aimed to identify clinical and anthropometric predictors of mortality among infants admitted with RSV pneumonia and to assess changes in mortality over a 25-year surveillance period.

**Methods:**

In this retrospective cohort study, we analysed 25 years of paediatric surveillance at Kilifi County Hospital, Kilifi, Kenya, spanning 25 successive RSV seasons. Neonates (aged <28 days) and post-neonatal infants (aged 28 days to 12 months) admitted with WHO-defined pneumonia were tested for RSV and demographic, anthropometric, and clinical data were recorded at admission. The primary outcome was in-hospital death among infants admitted with RSV pneumonia. Predictors of mortality were identified using random-forest and multivariable logistic regression, and temporal trends in mortality and anthropometric status were examined.

**Findings:**

Of 75 482 admissions of infants to Kilifi County Hospital between Jan 1, 2001, and July 13, 2025, 19 299 (25·6%) met WHO pneumonia criteria and 2745 (22·7%) had RSV. Of these infants, 2390 (87·1%) were post-neonatal, of whom 58 (2·4%) died in hospital with 44 (76%) deaths within 7 days of admission. Mortality was independently associated with congenital heart disease (odds ratio 3·51 [95% CI 1·37–8·97]), severe undernutrition (per 1-unit reduction in weight-for-age Z score: 1·59 [1·28–1·99]), and hypoxaemia (per 1% decrease in peripheral oxygen saturation: 1·05 [1·03–1·08]). 38 (70·4%) of 54 infants with RSV who died in hospital had a mid-upper arm circumference below the severe acute malnutrition threshold of 11·5 cm. There was no evidence of a sustained decline in RSV-associated in-hospital mortality or improvement in anthropometric status over the 25-year study period.

**Interpretation:**

RSV mortality in Kenyan infants remains high and has not declined over 25 years. Severe undernutrition and congenital heart disease identify infants with RSV who are at the highest risk of in-hospital death. These findings highlight the role of chronic anthropometric deficits in RSV outcomes and support targeted nutritional interventions to reduce mortality.

**Funding:**

Bill & Melinda Gates Foundation and Wellcome.

## Introduction

Respiratory syncytial virus (RSV) is the leading cause of acute lower respiratory infection in young children worldwide. Global estimates suggest that RSV accounts for 3·6 million hospitalisations annually^1^ and over 100 000 deaths in children younger than 5 years.^1^ The mortality burden is borne disproportionately by low-income and middle-income countries (LMICs),^2^^,^^3^ which account for more than 95% of RSV-related paediatric deaths,^1^ with infants in the first year of life bearing the greatest burden of disease. The licensure of maternal vaccines and long-acting monoclonal antibodies^4^^,^^5^ represents a major advance in RSV prevention, but their potential to reduce infant mortality will depend on how well they are targeted to the populations at highest risk. Most detailed data on RSV mortality in infants are derived from high-income countries (HICs), where risk profiles might not be fully generalisable to lower-resource settings.^6^

**Research in context**

**Evidence before this study**

We searched PubMed using the terms (“respiratory syncytial virus” OR “RSV”) AND (“mortality” OR “risk factors”) AND (“infants” OR “children”) AND (“Africa”), and included studies published from Jan 1, 1999, to June 30, 2025, with no language restrictions. Most studies were short-term investigations spanning only 1–5 years and typically included fewer than 100 infants or children positive for respiratory syncytial virus (RSV). Studies from The Gambia, South Africa, and Tunisia between 1999 and 2009 described the epidemiology of RSV hospitalisation and identified young age and HIV infection as risk factors for severe disease. Later studies conducted between 2010 and 2019 from Mozambique and multicountry studies, such as PERCH, expanded the aetiological understanding of pneumonia in African children, but seldom reported mortality-specific analyses. Between 2017 and 2023, large multinational collaborations within the RSV GOLD network have substantially advanced understanding of global RSV mortality patterns, including analyses of community infant deaths across multiple low-income and middle-income countries (LMICs), nosocomial RSV mortality, and prospective cohorts from intensive care units in countries eligible for Gavi, the Vaccine Alliance. Collectively, these studies have shown that most RSV-related deaths occur in early infancy and often outside tertiary care settings, and have characterised age distributions and comorbid conditions among fatal cases. However, these analyses were largely multicountry, registry-based cohorts and did not evaluate long-term temporal trends within a defined population or systematically quantify anthropometric nutritional status as a graded mortality risk factor.

**Added value of this study**

This 25-year study represents one of the largest RSV surveillance datasets globally and the most detailed analysis of mortality risk in Kenyan infants. Using random-forest classification and multivariable regression, we identified congenital heart disease, hypoxaemia, and severe undernutrition as the dominant predictors of in-hospital death. We show a graded association between worsening anthropometric status and RSV mortality risk, and show that mortality risk persists beyond hospital discharge, with substantial post-discharge deaths occurring within 90 days. The extended temporal span allows for the assessment of mortality patterns over multiple decades under routine-care conditions in an LMIC setting, offering an analysis of long-term trends not achievable in shorter-term studies. Importantly, we found no evidence of a sustained decline in RSV mortality or improvement in anthropometric risk factors over the 25-year surveillance period, indicating that RSV mortality remains a persistent problem in this setting.

**Implications of all the available evidence**

Our findings indicate that although RSV prophylaxis strategies remain central to global prevention, reducing mortality in LMICs will also require context-specific interventions that address undernutrition as an underlying risk factor for RSV mortality. The absence of improvement in RSV mortality and anthropometric risk factors over more than two decades highlights the need for renewed focus on structural and nutritional determinants of child health. Systematic nutritional screening and early post-discharge support for infants at high risk could meaningfully reduce RSV-related deaths in high-burden African settings.

In HICs, RSV deaths are rare and are largely confined to infants with inherent biological vulnerabilities such as congenital heart disease,^7^^,^^8^ chronic lung disease,^9^^,^^10^ or premature birth.^11^^,^^12^ In many LMICs, health-system constraints, including delays in seeking care, insufficient oxygen delivery, and restricted paediatric intensive care capacity, increase the risk that otherwise survivable RSV episodes become fatal. Children might also present with underlying biological vulnerabilities that further elevate mortality risk, even with timely hospital access.^13^^,^^14^ Some of these risk factors might be modifiable, underscoring the importance of defining their contribution in high-burden settings.

To address this question, we analysed 25 years of retrospectively collected surveillance data from a large paediatric cohort in rural Kenya. By linking routine clinical assessments, anthropometry, and laboratory diagnoses with detailed outcome data, we aimed to identify locally relevant predictors of mortality among infants with RSV admitted to a rural Kenyan hospital.

## Methods

### Study design and participants

This retrospective cohort study was conducted at Kilifi County Hospital (KCH), a large referral facility on the north coast of Kenya that serves a predominantly rural population of approximately 300 000 people.^15^ Since 2001, the Kenya Medical Research Institute (KEMRI)–Wellcome Trust Research Programme has maintained continuous surveillance of admissions of paediatric acute respiratory infection at KCH.

We performed a retrospective analysis of infants with RSV admitted to KCH between Jan 1, 2001, and July 13, 2025, within an ongoing hospital-based surveillance platform in Kilifi, Kenya. Infants were screened with WHO Integrated Management of Childhood Illness (IMCI) criteria, and eligible participants were those admitted with WHO-defined pneumonia who tested positive for RSV on upper respiratory sampling. The primary analysis was restricted to post-neonatal infants (aged ≥28 days to 12 months), with neonatal infants (aged <28 days) analysed separately. Written informed consent was obtained from the parents and legal guardians of all infants. The study was approved by KEMRI Scientific and Ethical Review Unit (number 3178) and conducted in accordance with Good Clinical Laboratory Practice principles. All data were anonymised and delinked from personal identifiers.

### Procedures

At admission, study clinicians used a structured electronic case report form to record clinical history, sex, vital signs, and anthropometric measurements; upper respiratory samples were obtained from all eligible infants for respiratory pathogen testing. RSV diagnoses were performed with immunofluorescent antibody testing between 2001 and 2009, and by multiplex real-time PCR in combination with immunofluorescent antibody testing from 2010 onwards (appendix 2 pp 1–2). Baseline variables included demographic factors (eg, age, sex, and location of residence), anthropometry (eg, weight-for-age Z score and mid-upper arm circumference [MUAC]), and clinical indicators of disease severity (eg, respiratory rate, temperature, oxygen saturation, haematology, and signs of respiratory distress). Anthropometric measurements were obtained at admission as part of routine IMCI-based assessments to identify severe acute malnutrition. HIV testing was performed at admission with parental or caregiver consent. Information on comorbid conditions, including congenital heart disease, was obtained from echocardiography and medical records.

### Statistical analysis

The primary outcome was in-hospital death among post-neonatal infants admitted with RSV pneumonia. To examine whether in-hospital mortality or nutritional status at presentation changed over the 25-year surveillance period, we did temporal trend analyses among all post-neonatal infants positive for RSV. The calendar year of admission was modelled as a continuous variable. Trends in in-hospital mortality were assessed with logistic regression with death during admission as the outcome, adjusting for age at admission. The presence of a temporal trend was established by the coefficient for calendar year, in which a non-significant coefficient was interpreted as no evidence of a change in mortality over time. Changes in anthropometric status over time were similarly evaluated with linear regression models with weight-for-age Z score and MUAC as continuous outcomes, also adjusting for age at admission. For visualisation, annual summary measures were calculated for each calendar year. In-hospital mortality was expressed as the annual case-fatality rate, defined as the number of RSV-positive in-hospital deaths divided by the total number of RSV-positive admissions in that year. Similarly, the prevalence of severe undernutrition was calculated annually with weight-for-age Z score (−3 or lower) and MUAC (<11·5 cm). These annual estimates were plotted as observed values for each year of surveillance. Random-forest analysis was used as a data-guided approach to rank candidate predictors of mortality based on their relative importance to support variable down selection for subsequent regression modelling. The dataset was randomly partitioned into training (60%) and validation (40%) subsets. This split was selected to balance stable model development with reliable performance evaluation, given the relatively small number of deaths. A 60–40 partition retained sufficient outcome events in the training set to allow robust variable selection and model fitting while preserving an adequately sized validation cohort to enable meaningful assessment of discrimination and calibration. Random-forest models were implemented in R (randomForest package) with a classification framework with 500 decision trees. At each split in the trees, a random subset of candidate predictors was considered. The size of this subset was selected by evaluating values around the standard default using out-of-bag error within the training set, with an optimal value of three predictors (from 35 total predictors) identified. Trees were grown to full depth with bootstrap sampling. Variable importance was quantified by the change in classification accuracy when each variable was permuted. Model performance was assessed in the validation set with area under the receiver operating characteristic curve, sensitivity, and specificity, and interpreted relative to a non-informative baseline (area under the curve 0·5) and a majority-class classifier reflecting outcome imbalance. Variables with more than 30% missingness were excluded from the analysis. To obtain effect-size estimates for the most influential predictors, multivariable logistic-regression models were fitted with in-hospital mortality as the dependent variable, with all models adjusted for age and sex. We conducted time-to-event analyses using the Kaplan–Meier analysis, with differences assessed using log-rank tests. For participants who were RSV positive and survived to discharge, survival after discharge was analysed separately, with follow-up from discharge to death or censoring at 365 days. Survival was compared across the same risk groups used for in-hospital analyses, and probabilities at 90 days and 365 days were summarised. Wilcoxon rank-sum tests were used to compare nutritional indices (ie, weight-for-age Z score and MUAC) at discharge between participants who died and those who survived to 12 months. All analyses were conducted in R (version 4.4.3).

### Role of the funding source

The funders of the study had no role in study design, data collection, data analysis, data interpretation, or writing of the report.

## Results

**Figure 1:**
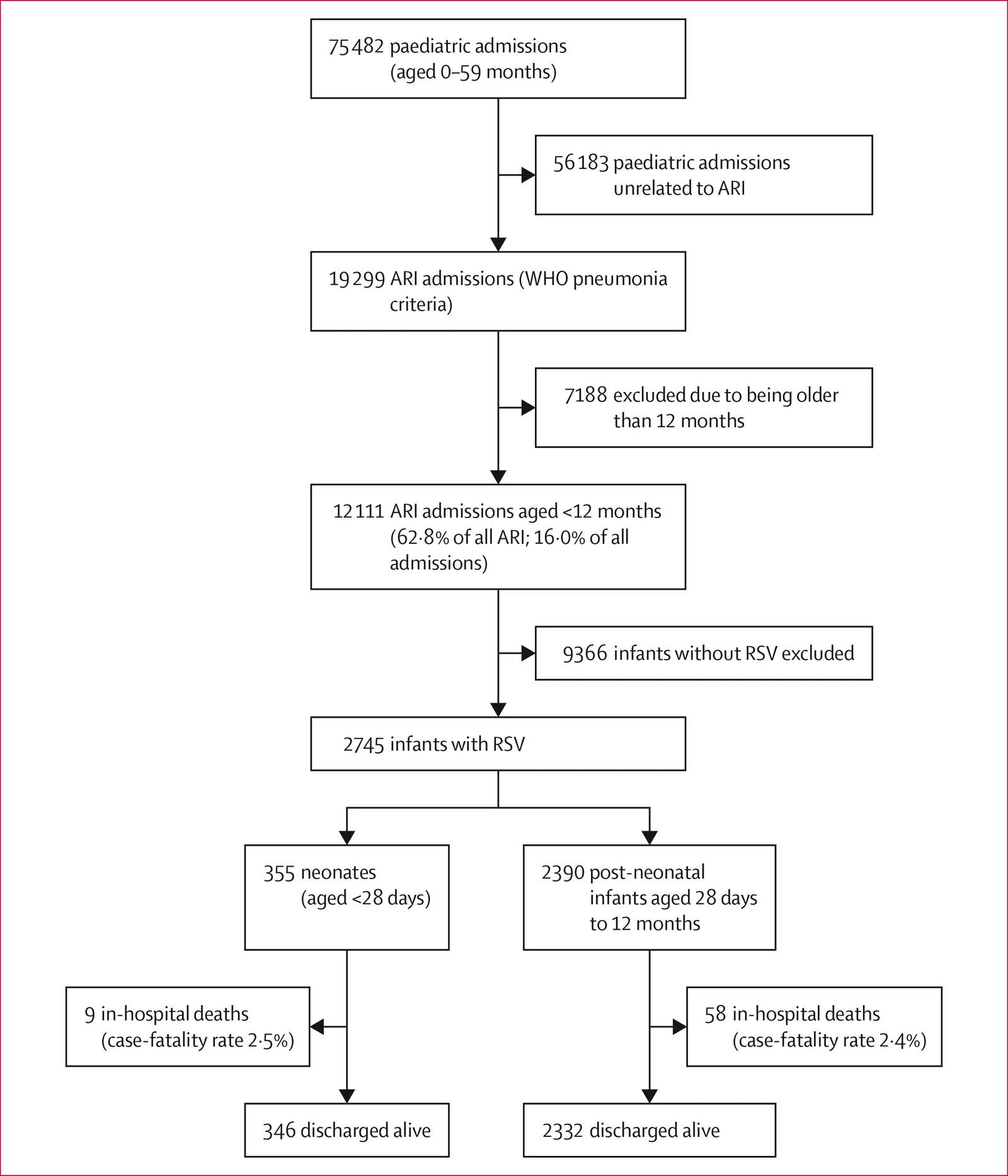
Trial profile ARI=acute respiratory illness. RSV=respiratory syncytial virus.

Between Jan 1, 2001, and July 13, 2025, 75 482 paediatric admissions (aged 0–59 months) were recorded at KCH, of whom 19 299 (25·6%) met WHO syndromic criteria for pneumonia. Infants younger than 12 months accounted for 12 111 (62·8%) pneumonia admissions and 16·0% of all paediatric admissions (figure 1). Among infants with pneumonia, 2745 (22·7%) tested positive for RSV: 355 (12·9%) were neonates (aged <28 days); 2390 (87·1%) were post-neonatal infants (aged 28 days to 12 months), forming the cohort for main primary analysis. The median age at admission was 3·9 months (IQR 2·1–6·1), and 1681 (70·3%) of 2390 were younger than 6 months. Male infants accounted for 1364 (57·1%) admissions. Baseline demographic and clinical features are summarised in the table and the appendix 2 (pp 3–6). In-hospital mortality among infants who were post-neonatal was 2·4% (58 of 2390), corresponding to 24 deaths per 1000 admissions; 2332 (97·6%) were discharged alive. Among the 355 neonates, nine (3%) in-hospital deaths occurred.

**Table:** Demographic, clinical, and anthropometric characteristics of the cohort

	Overall (N=2390)	Survivors (n=2332)	Deaths (n=58)
Age, months	3·9 (2·1–6·7)	3·9 (2·1–6·7)	4. 5 (2·6–8·4)
Age group			
<6 months	1681 (70·3%)	1642 (70·4%)	39 (67·2%)
6–12 months	709 (29·7%)	690 (29·6%)	19 (32·8%)
Sex			
Female	1026 (42·9%)	993 (42·6%)	33 (56·9%)
Male	1364 (57·1%)	1339 (57·4%)	25 (43·1%)
Pneumonia status			
Very severe	350 (14·6%)	320 (13·7%)	30 (51·7%)
Severe	1957 (81·9%)	1930 (82·8%)	27 (46·6%)
Mild	38 (1·6%)	38 (1·6%)	0
None∗	45 (1·9%)	44 (1·9%)	1 (1·7%)
Congenital heart disease			
Yes	37 (1·5%)	27 (1·2%)	10 (17·2%)
No	2353 (98·5%)	2305 (98·8%)	48 (82·8%)
Weight-for-age Z score	–0·6 (1·5)	–0·6 (1·4)	–2·3 (1·7)
Mid-upper arm circumference, cm	12·5 (1·7)	12·6 (1·7)	10·5 (2·0)
Respiratory rate per min	58·9 (14·1)	58·8 (14·1)	61·9 (16·5)
Temperature, °C	37·7 (1·0)	37·7 (1·0)	37·7 (1·3)
Oxygen saturation	94·4% (7·3)	94·5% (7·0)	87·5% (13·9)

Data are n (%), median IQR, and mean (SD).

∗ Several infants with RSV were classified as having no pneumonia according to WHO Integrated Management of Childhood Illness criteria. These infants presented with severe respiratory symptoms but did not meet the full algorithmic criteria for pneumonia and were admitted for management.

**Figure 2:**
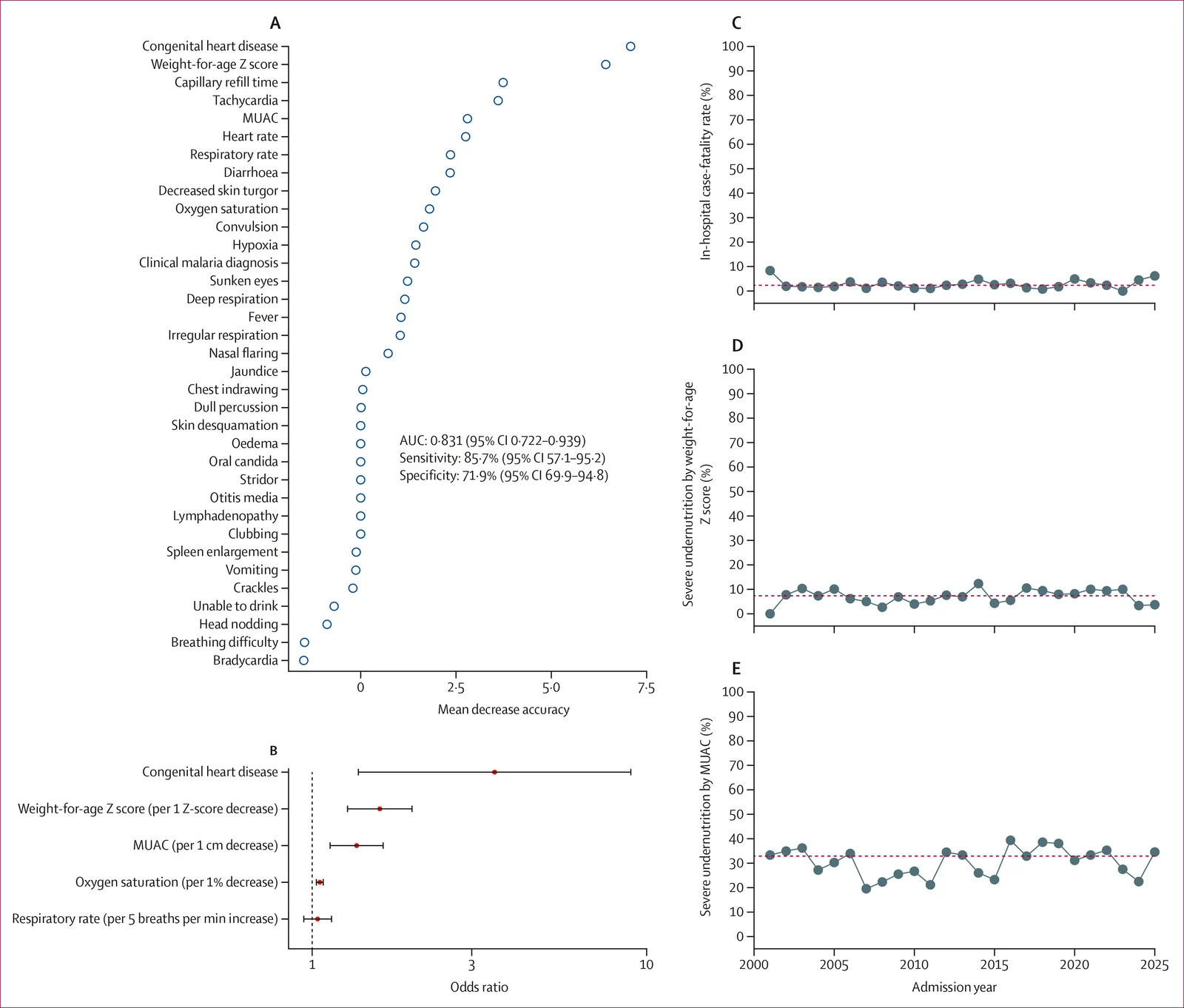
Predictors and temporal trends of RSV mortality among post-neonatal infants (A) Random-forest variable importance plot showing predictors of in-hospital mortality among post-neonatal infants with RSV. Model performance is shown inset. (B) Multivariable logistic regression estimates (odds ratios and 95% CIs) for selected predictors of in-hospital mortality. Decreasing congenital heart disease, weight-for-age Z score (per 1 decrease in Z score), decreasing MUAC (per 1 cm decrease), lower oxygen saturation (per 1% decrease), and higher respiratory rate (per 5 breaths per min increase) were associated with increased odds of in-hospital death. (C–E) Temporal trends over the 25-year surveillance period among post-neonatal infants with RSV. (C) Annual crude in-hospital case-fatality rate. (D) Proportion with severe undernutrition defined as a weight-for-age Z score −3 or lower. (E) Proportion with severe undernutrition defined as a MUAC of 11·5 cm or lower. Points represent annual proportions. AUC=area under the curve. MUAC=mid-upper arm circumference. RSV=respiratory syncytial virus.

RSV contributed substantially to paediatric respiratory admissions across those younger than 5 years (appendix 2 p 7), with peak prevalence in early infancy. The highest admission prevalence occurred in the second month of life, during which 36·8% of all pneumonia admissions were RSV positive. Among the 58 post-neonatal infants who died, mortality occurred predominantly early in the admission course: 14 (24%) died within 24 h, 31 (53%) within 72 h, and 44 (76%) within the first week of hospitalisation. To identify predictors of mortality among post-neonatal infants admitted with severe RSV, we first applied random-forest-based machine learning approaches. In the random-forest model, congenital heart disease, severe undernutrition (measured with weight-for-age Z score and MUAC), reduced oxygen saturation, and clinical signs of poor perfusion (reduced capillary refill time and decreased skin turgor) ranked as the strongest predictors of in-hospital mortality (figure 2A). The model achieved an area under the receiver operating characteristic curve of 0·831 (95% CI 0·722–0·939; appendix 2 pp 8–9), with a sensitivity of 85·7% (95% CI 57·1–95·2) and specificity of 71·9% (95% CI 69·9–94·8; figure 2A).

We then applied multivariable logistic regression to obtain effect size estimates. Congenital heart disease was associated with substantially increased odds of death (odds ratio 3·51 [95% CI 1·37–8·97]; p=0·015). Weight-for-age Z score showed a similarly strong effect: each Z-score decrease of 1 was associated with a 59% increase in the odds of death (1·59 [1·28–1·99]; p<0·0001). Reduced blood oxygen was also independently associated with mortality, with each 1% decrease in blood oxygen saturation conferring an increased mortality risk (1·05 [1·03–1·08; p<0·0001). Increased respiratory rate was not independently associated with RSV mortality (figure 2B). We examined whether mortality or nutritional status at presentation changed over the 25-year surveillance period. Annual case-fatality rates fluctuated modestly from year to year but showed no evidence of a sustained temporal decline (figure 2C). In age-adjusted logistic regression, calendar year was not associated with in-hospital mortality (1·03 [0·9–1·06]; p=0·11), indicating no evidence of a temporal trend over the study period. Similarly, anthropometric status at admission did not show progressive improvement over time (figure 2D, E). In linear regression models adjusted for age at admission, there was no significant association between calendar year and weight-for-age Z score (*β*=0·003, p=0·49) or MUAC (*β*=−0·007, p=0·19). HIV infection was uncommon among infants with RSV in this cohort. Of 2390 post-neonatal infants who were RSV positive, 47 (2·0%) were HIV positive. Five (10·6%) deaths occurred among infants with HIV with RSV compared with 53 (2·3%) deaths among 2343 infants without HIV. Overall, infants with HIV accounted for five (8·6%) of 58 in-hospital RSV deaths. Overall, HIV prevalence among infants positive for RSV was low across the study period and showed no consistent temporal trend (appendix 2 p 10).

**Figure 3:**
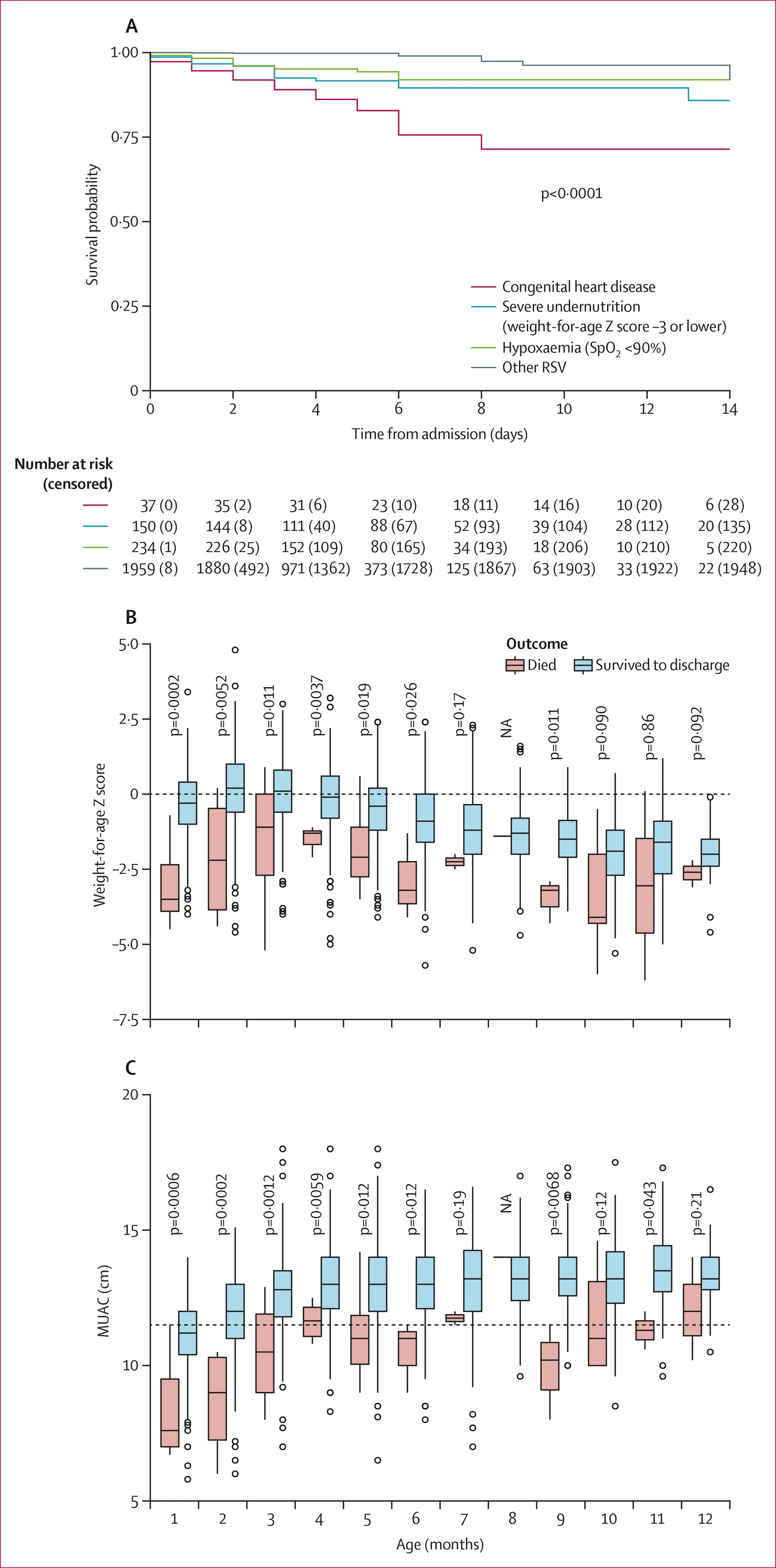
Early in-hospital mortality and age-stratified anthropometric differences among post-neonatal infants with RSV (A) Kaplan–Meier curves showing 14-day in-hospital survival among post-neonatal infants with RSV with complete outcome data (n=2381), stratified by congenital heart disease, severe undernutrition, hypoxaemia, and infants without these risk factors (ie, other RSV). Mortality was concentrated within the first week of admission and was highest among infants with congenital heart disease and severe undernutrition (log-rank p<0·0001). (B) Weight-for-age Z scores at admission among infants with RSV who died versus those who survived to discharge, stratified by month of post-neonatal life (1–12 months). The horizontal dashed line indicates a weight-for-age Z score of zero. (C) MUAC at admission among infants who died versus those who survived to discharge, stratified by month of post-neonatal life. The horizontal dashed line indicates a MUAC severe undernutrition threshold of 11·5 cm. p values represent Wilcoxon rank-sum tests comparing infants who died with those who survived within each age stratum. MUAC=mid-upper arm circumference. NA=not applicable. RSV=respiratory syncytial virus.

**Figure 4:**
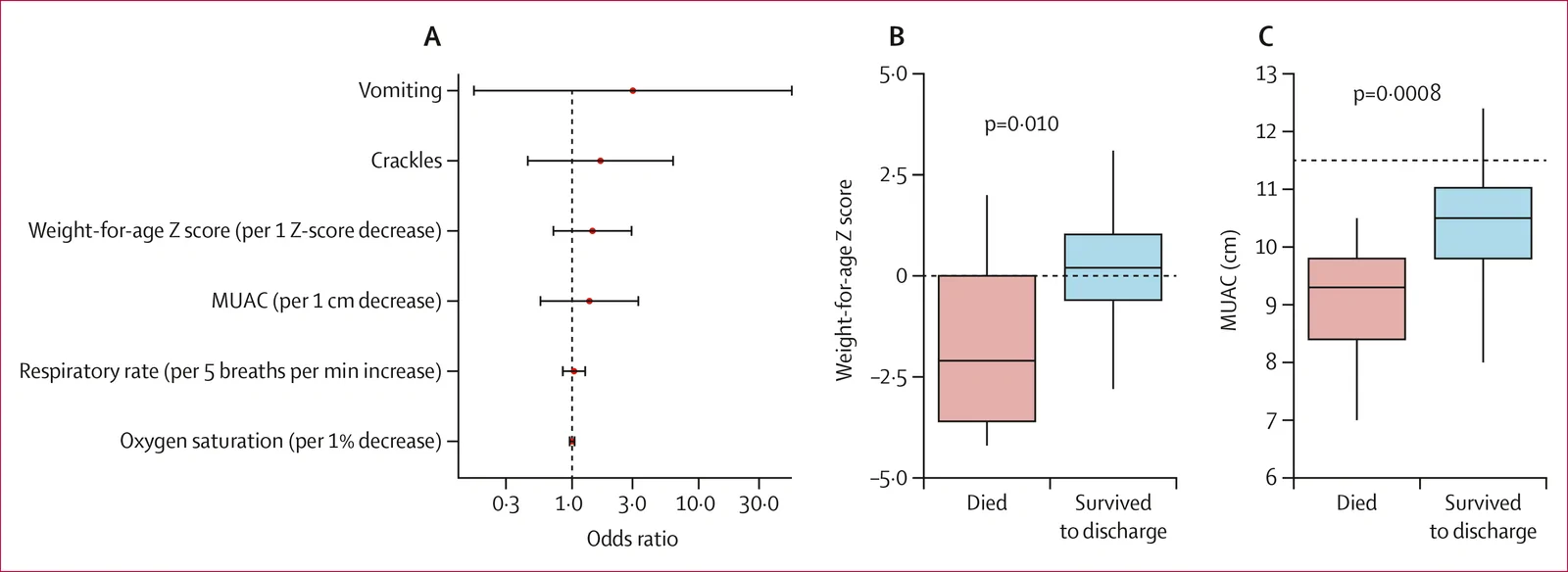
Predictors of in-hospital mortality among neonates (aged <28 days) with respiratory syncytial virus (A) Multivariable logistic regression estimates (odds ratios and 95% CIs) for selected predictors of neonatal in-hospital mortality. Vomiting, crackles, decreasing weight-for-age Z score (per 1 Z-score decrease), decreasing MUAC (per 1 cm decrease), higher respiratory rate (per 5 breaths per min increase), and lower oxygen saturation (per 1% decrease) were associated with higher odds of death. However, confidence intervals were wide and crossed unity for all predictors. (B) Admission weight-for-age Z scores among neonates who died versus those who survived to discharge. The horizontal dashed line indicates a weight-for-age Z score of zero. (C) Admission MUAC among neonates who died versus those who survived to discharge. The horizontal dashed line indicates a MUAC severe malnutrition threshold of 11·5 cm. Boxplots represent median and IQR; p values are derived from Wilcoxon rank-sum tests. MUAC=mid-upper arm circumference.

Kaplan–Meier curves stratified by major risk groups showed clear and early divergence (figure 3A). Infants with congenital heart disease had the highest mortality, with a 14-day post-admission survival of 70·1% (95% CI 54·3–90·5). Severe undernutrition, defined as a weight-for-age Z score of –3 or lower, was the next strongest discriminator, with a survival of 84·5% (74·9–95·2) by day 14. Infants with hypoxaemia (peripheral oxygen saturation <90%) had intermediate outcomes, with a day 14 survival of 91% (86·2–96·1). Infants without these major risk factors had a day-14 survival of 90% (81·6–100·0; figure 3A). Anthropometric deficits were consistently observed among infants with RSV who died, irrespective of age. Infants with RSV who died had significantly lower weight-for-age Z scores and MUAC compared with survivors (figure 3B, C; appendix 2 p 11). These differences were evident at each month of post-neonatal infancy, with no evidence that the difference in these anthropometric measurements were attenuated with increasing age (figure 3B, C). Nearly all infants who died had MUAC values below the severe malnutrition MUAC threshold of 11·5 cm (38 [70·4%] of 54 whose admission MUACs were available), whereas most survivors (1623 [75·6%] of 2146 for whom MUACs were available) were above this threshold (figure 3C). To explore predictors of mortality in neonates, we conducted secondary analyses restricted to infants with RSV younger than 28 days (figure 4). In a random-forest analysis, clinical signs of respiratory distress and anthropometric vulnerability ranked the highest in importance for mortality classification, including crackles, vomiting, lower weight-for-age Z score, and lower MUAC (appendix 2 p 12). In multivariable logistic regression, the direction of association was consistent with these findings. Vomiting, crackles, higher respiratory rate, and lower oxygen saturation were associated with higher odds of in-hospital death. Similarly, decreasing weight-for-age Z score and decreasing MUAC were associated with higher odds of mortality (figure 4B). However, confidence intervals were wide and crossed unity for all predictors. In comparative analysis, RSV-infected neonates who died had significantly lower admission weight-for-age Z scores and MUAC compared with those who survived to discharge (figure 4C).

**Figure 5:**
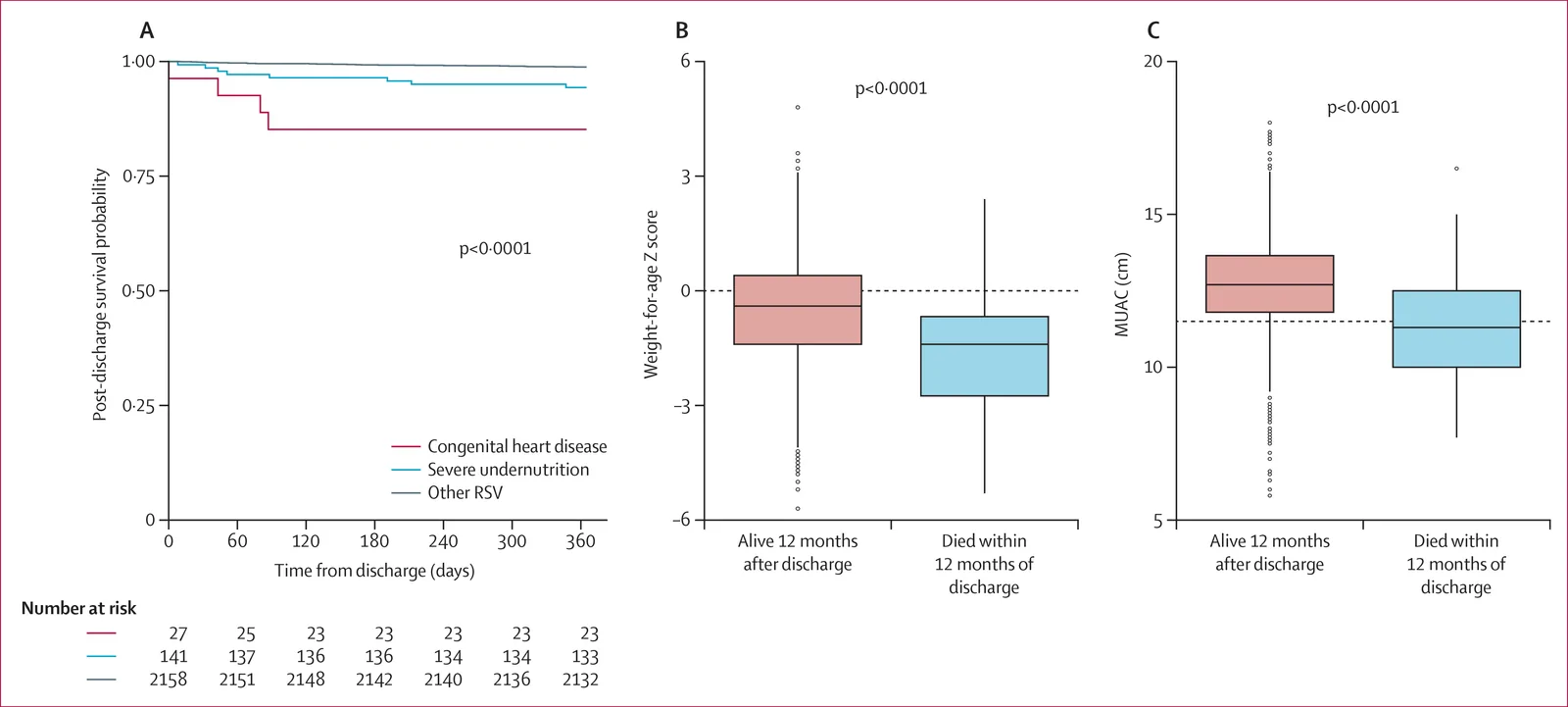
Post-discharge survival and discharge anthropometry among post-neonatal infants (aged ≥28 days) with RSV (A) Kaplan–Meier curves showing 12-month post-discharge survival among infants with RSV with complete follow-up data (n=2326), stratified by congenital heart disease, severe undernutrition defined as weight-for-age Z score of −3 or less, and other infants with RSV without these risk factors. Infants with congenital heart disease or severe undernutrition had significantly lower 12-month survival probabilities (log-rank p<0·0001). No participants included in this analysis were censored before 12 months. (B) Weight-for-age Z score at discharge among children who died within 12 months of discharge compared with those who survived to 12 months. The p value was derived with a Wilcoxon rank-sum test. (C) MUAC at discharge among children who died within 12 months of discharge compared with those who survived to 12 months. The p value was derived with a Wilcoxon rank-sum test. MUAC=mid-upper arm circumference. RSV=respiratory syncytial virus.

Next, we examined whether risk factors associated with in-hospital death were also linked to long term outcomes. Of the 2332 infants who were RSV positive and post-neonatal that were discharged, follow-up over a period of 12 months after discharge revealed that mortality risk persisted beyond discharge. Kaplan–Meier analysis for over 12 months showed that deaths after discharge occurred predominantly within the first 90 days (figure 5A). By 12 months, estimated survival probabilities were 85·2% (95% CI 72·8–99·7) among infants with congenital heart disease, 94·3% (90·6–98·2) among infants with undernourishment, and 98·8% (98·3–99·3) among all other infants with RSV. Among the infants who were RSV positive and post-neonatal, 58 (24·9%) deaths occurred during hospitalisation and 38 (16·3%) occurred within 12 months of discharge, yielding 96 total deaths. Thus, post-discharge deaths accounted for 38 (39·6%) of 96 deaths. Anthropometric indicators at discharge strongly predicted post-discharge outcome (figure 5B, C). Infants who died within 12 months of discharge had significantly lower weight-for-age Z scores and smaller mid-upper arm circumference than those who survived.

Finally, as a post-hoc analysis, we examined whether in-hospital mortality estimates might have been influenced by interruptions in public health-care services over the 25-year surveillance period or the COVID-19 pandemic. Weekly aggregation of paediatric pneumonia admissions showed sustained recruitment throughout the study period, with no cessation of enrolment or diagnostic testing, including during periods of public health-care worker strikes and the COVID-19 pandemic (appendix 2 pp 13–14). 25 successive RSV seasons were detected over the 25 years of surveillance and there was no evidence of systematic suppression of case ascertainment over that time.

## Discussion

In this retrospective cohort study with 25 years of continuous surveillance, in-hospital mortality among infants admitted with RSV pneumonia in rural Kenya was 2·4%, corresponding to 24 deaths per 1000 admissions. This finding remains substantially higher than estimates from HICs, where RSV case-fatality among hospitalised infants is typically below 0·5%.^1^ The contrast reflects the disproportionate global burden of RSV mortality, with most paediatric deaths occurring in LMICs. Although infection incidence can be broadly similar across settings, fatal outcomes are concentrated in LMIC settings, as shown in the RSV GOLD study, which showed that mortality largely occurs in early infancy for severe RSV infection acquired in the community and nosocomially^12^^,^^16^, ^17^ and RSV-related deaths that occur in the community.^3^

In HICs, determinants of RSV mortality are well characterised. These insights informed targeted prophylaxis with palivizumab for infants at high risk.^18^, ^19^, ^20^ However, risk profiles are less defined in LMICs than in HICs, and extrapolation from HIC settings could overlook locally relevant vulnerabilities. Our findings supported congenital heart disease as an important predictor of mortality, but the more striking observation was the prominence of undernutrition as a predictor of mortality. Anthropometric deficits are uncommon in HIC cohorts but widespread in LMICs, affecting an estimated 165 million children younger than 5 years.^21^ Although congenital anomalies represent a relatively small high-risk subgroup, nutritional vulnerability affects a far larger proportion of infants. We found that each 1-unit decrease in weight-for-age Z score was associated with a 59% increase in the odds of death. Nearly all non-survivors had MUAC values below the WHO threshold for severe acute malnutrition (11·5 cm).^22^^,^^23^ This pattern was consistent across infancy and across anthropometric measures, suggesting that chronic nutritional vulnerability is a central determinant of RSV mortality in this setting.

The primary analysis focused on post-neonatal infants, with secondary analyses examining neonates. The neonatal period is characterised by heterogeneous causes of severe illness, including sepsis and perinatal complications, impeding a definitive attribution of death to the detection of RSV alone. WHO IMCI frameworks distinguish neonatal illness from later infancy for this reason.^24^ In secondary neonatal analyses, anthropometric vulnerability showed similar directional associations with mortality, but confidence intervals were wide, reflecting the low numbers of events and the complexity of neonatal attribution. These findings highlight both the potential contribution of RSV in early life and the challenges of disentangling viral from concurrent neonatal pathology.

Across 25 years of surveillance, we found no sustained decline in in-hospital mortality. Age-adjusted analyses showed no significant temporal trend, and anthropometric status at admission did not show progressive improvement. These findings suggest that both mortality risk and nutritional vulnerability remained broadly stable over two decades. Although the capacity for supportive care has improved incrementally over time, these changes have not translated into measurable reductions in RSV mortality. Persistent underlying vulnerability, particularly severe undernutrition, could continue to shape outcomes. However, because of the low number of deaths, temporal analyses adjusted only for age and assumed independence of observations over time. Therefore, there is a possibility that recurrent RSV admissions and unmeasured changes in comorbidity profiles or clinical care might not have been fully captured. HIV prevalence among infants with RSV was low, and children with HIV accounted for a small proportion of deaths, which precluded a precise estimation of effect size. Although crude mortality was higher in infants with HIV, event numbers were low, which likely reflects a lower prevalence of infant HIV in coastal Kenya compared with southern African settings^25^^,^^26^ and the success of prevention of mother-to-child transmission programmes. A further limitation is that data on in-utero HIV exposure were not collected; therefore we were unable to identify infants who were exposed to HIV and uninfected, which is a group that might be at an increased risk of severe infection. The duration of symptoms before hospital presentation was also not systematically recorded and could not be analysed, despite possibly being an important predictor of mortality. In addition, data on other comorbidities were not consistently collected, restricting our ability to quantify the independent contribution of these risk factors in this population. We also excluded variables with substantial missingness in the random-forest modelling, rather than applying imputation methods, which could have limited the inclusion of potentially relevant predictors; future analyses could explore multiple imputation approaches. Tuberculosis is also an important cause of paediatric respiratory morbidity in sub-Saharan Africa. However, tuberculosis diagnostics were not systematically performed or recorded within this acute pneumonia surveillance platform over the 25-year period, precluding reliable assessment of tuberculosis co-infection in relation to RSV-associated mortality.

Follow-up after discharge revealed that mortality risk extended beyond hospitalisation. Although overall 1-year survival exceeded 98% in most infants, those with congenital heart disease or severe undernutrition remained at an elevated risk. Nearly all deaths after discharge occurred within 90 days, indicating a crucial early convalescent window. Similar patterns have been observed in other paediatric cohorts in Africa, where mortality after discharge accounts for a substantial proportion of infection-related deaths. However, a definitive attribution of these deaths to RSV is not possible; the Child Health And Mortality Prevention Surveillance post-mortem studies of community deaths show that most RSV-associated community deaths occur in the context of multiple conditions in the causal chain.^27^ As our study is hospital based, it does not capture deaths that occur before children reach hospital, and total population-level RSV mortality is therefore likely underestimated.

These findings suggest that reducing RSV mortality in LMICs will require more than prophylaxis alone. Maternal vaccination and long-acting monoclonal antibodies represent transformative advances in prevention, but their effect on mortality could be maximised when integrated with strategies addressing underlying host vulnerability. In high-burden settings, maternal vaccination is likely to confer broad population-level benefit, whereas long-acting monoclonal antibodies could complement protection among infants missed by antenatal services or with impaired transplacental antibody transfer. Our data suggest that the greatest mortality reductions could be achieved when preventive tools are combined with systematic identification and management of severe anthropometric vulnerability. Anthropometric measurements are routinely recorded in many hospital settings, but our findings indicate that undernutrition should be explicitly recognised as a high-risk state in RSV infection, triggering intensified monitoring, early nutritional support, and structured follow-up after discharge. Although these observational data cannot establish whether undernourished infants should be prioritised for monoclonal prophylaxis, they do identify a biologically vulnerable subgroup warranting evaluation in maternal vaccine effectiveness and policy studies. Severe malnutrition arises from complex structural determinants, including food insecurity, poverty, and health-system constraints. Although our data do not address upstream prevention strategies, they do show that within the clinical context, systematic identification and active management of undernutrition represent immediately actionable opportunities to reduce RSV-associated mortality in LMICs.

## Data sharing

De-identified individual participant data underlying the findings of this study, together with the data dictionary, will be made available upon reasonable written request to the Data Governance Committee of the KEMRI–Wellcome Trust Research Programme (datarequests@kemri-wellcome.org). Requests will be reviewed in accordance with the Programme’s data access policies and relevant institutional and national ethical guidelines. Personally identifiable information cannot be shared because of Kenyan data protection legislation and ethical restrictions.

## Declaration of interests

We declare no competing interests.

## References

[1] You L, Xin W, Dianna MB (2022). Global, regional, and national disease burden estimates of acute lower respiratory infections due to respiratory syncytial virus in children younger than 5 years in 2019: a systematic analysis. Lancet.

[2] Willemsen JE, Vernooij FS, Shaaban FL, Chikoti C, Bont LJ, Drylewicz J (2024). Characteristics of inpatient and outpatient respiratory syncytial virus mortality in Gavi-eligible countries. Vaccine X.

[3] Mazur NI, Löwensteyn YN, Willemsen JE (2021). Global respiratory syncytial virus-related infant community deaths. Clin Infect Dis.

[4] Sonia A-G, Narmeen M, María Isolina S-P (2024). Effectiveness and impact of universal prophylaxis with nirsevimab in infants against hospitalisation for respiratory syncytial virus in Galicia, Spain: initial results of a population-based longitudinal study. Lancet Infect Dis.

[5] Ricardo C, Pierre Yves B, Aurélie P (2024). Real-world effectiveness of nirsevimab immunisation against bronchiolitis in infants: a case–control study in Paris, France. Lancet Child Adolesc Health.

[6] Sini de Almeida R, Leite J, Atwell JE (2024). Respiratory syncytial virus burden in children under 2 years old in understudied areas worldwide: gap analysis of available evidence, 2012–2022. Front Pediatr.

[7] Vartiainen P, Jukarainen S, Rhedin SA (2023). Risk factors for severe respiratory syncytial virus infection during the first year of life: development and validation of a clinical prediction model. Lancet Digit Health.

[8] Pa Saidou C, Stephanie Wen Lan W, Steve C, Harry C, Rafael M, Harish N (2020). Acute lower respiratory infections associated with respiratory syncytial virus in children with underlying congenital heart disease: systematic review and meta-analysis. J Infect Dis.

[9] Eric AFS, Shabir AM, William JM (2023). Efficacy of nirsevimab against respiratory syncytial virus lower respiratory tract infections in preterm and term infants, and pharmacokinetic extrapolation to infants with congenital heart disease and chronic lung disease: a pooled analysis of randomised controlled trials. Lancet Child Adolesc Health.

[10] Dallagiacoma G, Lundholm C, Smew AI (2025). Risk factors for severe outcomes of respiratory syncytial virus infection in children: a nationwide cohort study in Sweden. Lancet Reg Health Eur.

[11] Deng S, Cong B, Edgoose M, De Wit F, Nair H, Li Y (2024). Risk factors for respiratory syncytial virus-associated acute lower respiratory infection in children under 5 years: an updated systematic review and meta-analysis. Int J Infect Dis.

[12] Scheltema NM, Gentile A, Lucion F (2017). Global respiratory syncytial virus-associated mortality in young children (RSV GOLD): a retrospective case series. Lancet Glob Health.

[13] Salima K, Seema L, Maram A, Ghazala R, Salman K, Eva O (2025). Developmental health and vulnerability among young children in Pakistan: findings from a large-scale early childhood development assessment in Karachi. Early Child Res Q.

[14] Abdelrahman DN, Abdullahi FL, Abdu-Raheem F (2024). Respiratory syncytial virus infection among children younger than 2 years admitted to a paediatric intensive care unit with extended severe acute respiratory infection in ten Gavi-eligible countries: the RSV GOLD-ICU Network study. Lancet Glob Health.

[15] Scott JAG, Evasius B, Jennifer CM (2012). Profile: the Kilifi Health and Demographic Surveillance System (KHDSS). Int J Epidemiol.

[16] Löwensteyn YN, Willemsen JE, Mazur NI (2023). Nosocomial RSV-related in-hospital mortality in children <5 years: a global case series. Pediatr Infect Dis J.

[17] Xin W, You L, Ting S (2024). Global disease burden of and risk factors for acute lower respiratory infections caused by respiratory syncytial virus in preterm infants and young children in 2019: a systematic review and meta-analysis of aggregated and individual participant data. Lancet.

[18] von Lilienfeld-Toal M, Khawaja F, Compagno F (2025). Community-acquired respiratory virus infections in patients with haematological malignancies or undergoing haematopoietic cell transplantation: updated recommendations from the 10th European Conference on Infections in Leukaemia. Lancet Infect Dis.

[19] Ison MG, Hirsch HH (2019). Community-acquired respiratory viruses in transplant patients: diversity, impact, unmet clinical needs. Clin Microbiol Rev.

[20] Mori M, Yoshizaki K, Watabe S (2023). Safety, efficacy and pharmacokinetics of palivizumab in off-label neonates, infants, and young children at risk for serious respiratory syncytial virus infection: a multicenter phase II clinical trial. Lancet Reg Health West Pac.

[21] Black RE, Victora CG, Walker SP (2013). Maternal and child undernutrition and overweight in low-income and middle-income countries. Lancet.

[22] WHO (2023).

[23] Tahmeed A, Waseem A, Alemayehu A (2025). Infant-level and child-level predictors of mortality in low-resource settings: the WHO Child Mortality Risk Stratification Multi-Country Pooled Cohort. Lancet Glob Health.

[24] WHO (2013).

[25] Chanchal R, Gupta S, Kanta C, Singh K, Koonwar S (2019). Hypophosphataemia in severe acute malnutrition: a prospective observational study. Br J Nutr.

[26] Atkinson SH, Suchdev PS, Bode M (2025). Getting back on track to meet global anaemia reduction targets: a *Lancet Haematology* Commission. Lancet Haematol.

[27] Blau DM, Baillie VL, Els T (2021). Deaths attributed to respiratory syncytial virus in young children in high-mortality rate settings: report from Child Health And Mortality Prevention Surveillance (CHAMPS). Clin Infect Dis.

