# Supplementary material for: In-hospital and post-discharge mortality among infants with respiratory syncytial virus in rural Kenya: a 25-year retrospective cohort study
Source: Lancet Glob Health. 2026 Jul 10;14(9):None. doi: 10.1016/j.langlo.2026.104003 (PMC13541653; doi:10.1016/j.langlo.2026.104003)
Supplement: Supplementary file 1 [file mmc1.pdf]

THE LANCET
Global Health

Supplementary appendix 1

This translation in Kiswahili was submitted by the authors and we reproduce it as supplied. It has not been peer reviewed. The *Lancet's* editorial processes have only been applied to the original in English, which should serve as reference for this manuscript.

Tafsiri hii katika Kiswahili iliwasilishwa na waandishi na tunatengeneza tena kama hutolewa. Haijapitiwa. Mchakato wa hariri wa Lancet umetumika tu kwa asili kwa Kiingereza, ambayo inapaswa kutumika kama kumbukumbu kwa muswada hii.

Supplement to: Mburu MW, Nyadzua Katama E, Safari MS, et al. In-hospital and post-discharge mortality among infants with respiratory syncytial virus in rural Kenya: a 25-year retrospective cohort study. *Lancet Glob Health* 2026. https://doi.org/10.1016/j.langlo.2026.104003

Muhtasari

Utangulizi: Virusi vya Respiratory Syncytial (RSV) ndivyo chanzo kikuu cha kulazwa hospitalini kwa watoto wachanga kutokana na maambukizi ya nimonia duniani kote, ilhali zaidi ya 95% ya vifo hutokea katika nchi za kipato cha chini na cha kati. Vipimo vya kutabiri matokeo mabaya baada ya kulazwa hospitalini kutokana na RSV bado havijabainishwa vyema. Lengo letu lilikuwa kubainisha vipimo vya kiafya na vya kimaumbile vinavyotabiri vifo miongoni mwa watoto wachanga waliolazwa na homa ya mapafu inayosababishwa na RSV, na kutathmini mabadiliko ya vifo katika kipindi cha miaka 25 iliyopita.

Mbinu: Katika utafiti huu, tulichambua kumbukumbu za matibabu ya watoto waliolazwa katika Hospitali ya Kaunti ya Kilifi, Kenya, zilizojumuisha kipindi cha miaka 25 na misimu 25 mfululizo ya mikurupuko ya RSV. Watoto wachanga wenye umri wa chini ya siku 28 na watoto wenye umri wa siku 28 hadi miezi 12 waliolazwa na nimonia iliyotathminiwa kwa kutumia vigezo vya Shirika la Afya Ulimwenguni (WHO), walipimwa RSV, kisha takwimu za kidemografia, kimaumbile na kiafya zikarekodiwa wakati wa kulazwa. Lengo letu kuu lilikuwa kutathmini vifo vilivyotokea hospitalini miongoni mwa watoto waliolazwa na nimonia ya RSV. Vigezo vya utabiri wa vifo vilitambuliwa kwa kutumia mbinu ya 'random forest' na uchambuzi wa 'multivariable logistic regression'. Mielekeo ya muda katika vifo vilivyosababishwa na RSV, hali ya lishe, na ukuaji wa mwili kwa kipindi cha miaka 25 yalichunguzwa.

Matokeo:
Miongoni mwa watoto 75,482 waliolazwa katika Hospitali ya Kaunti ya Kilifi kati ya Januari 1, 2001, na Julai 13, 2025, watoto 19,299 (25.6%) walikidhi vigezo vya nimonia vya WHO, na 2,745 (22.7%) walikuwa na RSV. Kati ya watoto hawa, 2,390 (87.1%) walikuwa na umri wa kati ya siku 28 na miezi 12, ambapo 58 (2.4%) walifariki wakiwa hospitalini, huku vifo 44 (76%) vikitokea ndani ya siku 7 za kulazwa. Vifo vilihusishwa moja kwa moja na ugonjwa wa moyo wa kuzaliwa nao (uwiano wa uwezekano [OR] 3.51 [95% CI 1.37–8.97]), utapiamlo mkali (kwa kila kupungua kwa kipimo kimoja cha 'weight-for-age Z-score': 1.59 [1.28–1.99]), na upungufu mkubwa wa oksijeni kwenye damu (kwa kila kupungua kwa 1% ya kiwango cha oksijeni katika damu: 1.05 [1.03–1.08]). Kati ya watoto 54 wenye RSV waliofariki hospitalini, 38 (70.4%) walikuwa na mzingo wa mkono wa chini ya sentimeta 11.5, kiwango kinachoashiria utapiamlo mkali. Hakukuwa na ushahidi wa kupungua kwa vifo vinavyohusiana na RSV au kuboreka kwa hali ya lishe katika kipindi chote cha utafiti cha miaka 25.
Tafsiri: Vifo vinavyosababishwa na RSV miongoni mwa watoto nchini Kenya bado viko juu na havijapungua kwa miaka 25. Utapiamlo mkali na ugonjwa wa moyo wa kuzaliwa nao hubainisha watoto wenye RSV ambao wako katika hatari kubwa zaidi ya kufariki wakiwa hospitalini. Matokeo haya yanadhihirisha athari za muda mrefu za mapungufu ya ukuaji wa mwili katika matokeo ya ugonjwa wa RSV, na yanaonesha umuhimu wa afua za lishe zinazowalenga watoto waliolazwa na RSV ili kupunguza vifo.
