# Supplementary material for: In-hospital and post-discharge mortality among infants with respiratory syncytial virus in rural Kenya: a 25-year retrospective cohort study
Source: Lancet Glob Health. 2026 Jul 10;14(9):None. doi: 10.1016/j.langlo.2026.104003 (PMC13541653; doi:10.1016/j.langlo.2026.104003)
Supplement: Supplementary file 2 [file mmc2.pdf]

THE LANCET
Global Health

Supplementary appendix 2

This appendix formed part of the original submission and has been peer reviewed.
We post it as supplied by the authors.

Supplement to: Mburu MW, Nyadzua Katama E, Safari MS, et al. In-hospital and post-discharge mortality among infants with respiratory syncytial virus in rural Kenya: a 25-year retrospective cohort study. *Lancet Glob Health* 2026. https://doi.org/10.1016/j.langlo.2026.104003

Table of Contents

Supplementary Methods 1

Supplementary Results 2

Supplementary Table 1. Clinical characteristics of the cohort 3

Supplementary Table 2. Baseline haematological characteristics of the cohort 5

Supplementary Figure 1. RSV positive admissions 7

Supplementary Figure 2. Receiver operating characteristic curve......................... 8

Supplementary Figure 3. Random forest–predicted probabilities 9

Supplementary Table 3. HIV prevalence among RSV-positive infants.................. 10

Supplementary Table 4. Weight-for-age Z-scores for.. 11

Supplementary Table 5. MUAC for RSV survivors and non-survivors 11

Supplementary Figure 4. Random forest variable for RSV-positive neonates 12

Supplementary Figure 5. RSV seasonal epidemics.. 13

Supplementary Figure 6. Annual enrolment numbers.. 14

Supplementary Materials

Supplementary Methods

RSV Testing

From 2001 to 2009, the detection of RSV in respiratory samples was carried out using the Light Diagnostics RSV Direct Fluorescent Antibody (DFA) kit (Cat. No. 3125). This assay was performed according to the manufacturer's instructions. In brief, 200μL of the specimen was transferred onto a glass slide following centrifugation in a Cytospin 3 centrifuge (Thermo Shandon, UK) at 1000 rpm for 10 minutes. After centrifugation, cells were fixed in cold acetone and a single drop of DFA reagent was carefully applied to each well. The slides were then incubated at 37 °C for 30 minutes in a humidified moist chamber to ensure optimal binding conditions. Excess reagent was washed off in fresh phosphate-buffered saline, and the slides were subsequently allowed to air dry at room temperature. One drop of mounting fluid was applied to the centre of each well, and the preparations were examined under an epifluorescence microscope at magnifications ranging from X20 to X40. A positive RSV result was defined as the presence of epithelial cells displaying characteristic apple-green cytoplasmic fluorescence. To enhance accuracy and reproducibility, each slide was read by at least two independent trained observers, and discrepant results were resolved by a third tiebraking reader. From 2010, RSV detection was performed primarily through multiplex real-time PCR, which was implemented in addition to the IFAT assay to increase diagnostic sensitivity and specificity. RNA was extracted from 140 μL of nasopharyngeal and oropharyngeal swab suspension using the QIAamp Viral RNA Kit (Qiagen), according to the manufacturer's instructions. Reverse transcription and amplification were performed in a single step, targeting conserved regions of the RSV F, N and G genes. Amplification was carried out on the Applied Biosystems 7500 Real Time PCR platform, using cycling conditions of 50°C for 20minutes minutes for reverse transcription, followed by denaturation at 95 °C for 5 minutes, and 40 cycles of annealing and extension at 95 °C for 15 seconds and 60°C for 30 seconds respectively. Each run included both positive and negative controls. Positive controls consisted of RSV A2 cultured virus and cDNA from a previous low cycle threshold (Ct) RSV positive sample, whereas negative controls included nuclease free water and PCR primer mix only. Amplification curves were analysed automatically by the instrument software, with a Ct of ≤35 used to define positivity.

Clinical Chemistry and Haematology

At admission, venous blood samples were collected into orange top tubes containing heparin anticoagulant. A total volume of 2ml was obtained where possible. Following collection, samples were transported immediately to the clinical laboratory for processing. Plasma was separated by centrifugation at 4000 RPM (revolutions per minute)for 10 minutes within 30 minutes of collection. Biochemical assays were performed on the ILAB ARIES Clinical Chemistry System (Instrumentation Laboratory) using manufacturer-supplied reagents. The panel of analytes included alanine aminotransferase (ALT), aspartate aminotransferase (AST), alkaline phosphatase (ALP), γ-glutamyl transferase (GGT), bilirubin, albumin, creatinine, urea, sodium, potassium, calcium, magnesium, inorganic phosphate, and glucose. Measurements followed manufacturer protocols, with regular calibration against traceable standards. Complete blood counts (CBC) were performed using the AcT 5 diff analyser (Beckman Coulter). Parameters measured included total white blood cell count, haemoglobin, haematocrit, red blood cell counts, platelet count, and white cell counts. Calibration and maintenance were conducted in accordance with the manufacturer's instructions, and performance was benchmarked against United Kingdom National External Quality Assurance Service (UK NEQAS) external quality assurance scheme and the Royal College of Pathologists of Australasia (RCPA) for hematology and chemistry, respectively. The clinical laboratory adhered to Good Clinical Laboratory Practice (GCLP) standards. The laboratory also participated in international external quality assurance (EQA) programmes, including the UK NEQAS and RCPA, ensuring comparability of results with international standards. Instruments were covered under manufacturer service contracts, with preventive maintenance and calibration conducted on a scheduled basis.

Supplementary results

Of the 9 RSV-positive neonatal infants (<28 days) who died, 2 (22%) died within 24 h, 6 (67%) within 72 h, and 7 (78%) within the first week of hospitalisation.

Characteristic	Overall (N=2390)	Survivors (n=2332)	Deaths (n=58)
Capillary refill time (seconds)			
0	110(4.60)	109 (4.67)	1 (1.72)
1	1138(47.62)	1120 (48.03)	18 (31.03)
2	1108(46.36)	1078 (46.23)	30 (51.72)
3	33(1.38)	24 (1.03)	9 (15.52)
4	1(0.04)	1(0.04)	0
Tachycardia			
Yes	705(29.50)	685(29.37)	20(34.48)
No	1685(70.50)	1647(70.63)	38(65.52)
Heart rate, n (%)			
<80bpm	23(0.96)	22(0.94)	1(1.72)
80-160bpm	756(31.63)	732(31.39)	24(41.38)
161-180bpm	906(37.91)	893(38.29)	13(22.41)
181-200bpm	537(22.47)	524(22.47)	13(22.41)
>200bpm	166(6.95)	159(6.81)	7(12.07)
Diarrhoea			
Yes	296(12.38)	287(12.31)	9(15.52)
No	2094(87.62)	2045(87.69)	49(84.48)
Decreased skin turgor			
Yes	43(1.80)	38(1.63)	5(8.62)
No	2345(98.12)	2292(98.28)	53(91.38)
N/A	2(0.08)	2(0.09)	0
Convulsion			
Yes	67(2.80)	62(2.66)	5(8.62)
No	2311(96.70)	2259(96.87)	52(89.66)
N/A	12(0.50)	11(0.47)	1(1.72)
Hypoxia			
Yes	286(11.97)	262(11.23)	24(41.38)
No	2103(87.99)	2069(88.72)	34(58.62)
N/A	1(0.04)	1(0.04)	0
Clinical malaria diagnosis			
Malaria Positive	13(0.54)	12(0.51)	1(1.72)
Malaria Negative	2377(99.46)	2320(99.49)	57(98.28)
Sunken eyes			
Yes	68(2.85)	64(2.74)	4(6.90)
No	2320(97.07)	2266(97.17)	54(93.10)
N/A	2(0.08)	2(0.09)	0
Deep respiration			
Yes	404(16.90)	370(15.87)	34(58.62)
No	1986(83.10)	1962(84.13)	24(41.38)
Fever			
Yes	2063(86.32)	2013(86.32)	50(86.21)
No	327(13.68)	319(13.68)	8(13.79)

Irregular respiration			
Yes	167(6.99)	160(6.86)	7(12.07)
No	2221(92.93)	2170(93.05)	51(87.93)
N/A	2(0.08)	2(0.09)	0
Nasal flaring			
Yes	1536(64.27)	1494(64.07)	42(72.41)
No	853(35.69)	837(35.89)	16(27.59)
N/A	1(0.04)	1(0.04)	0
Jaundice			
Yes	5(0.21)	4(0.17)	1(1.72)
No	2385(99.79)	2328(99.83)	57(98.28)
Chest indrawing			
Yes	2293(95.94)	2237(95.93)	56(96.55)
No	96(4.02)	94(4.03)	2(3.45)
N/A	1(0.04)	1(0.04)	0
Dull percussion			
Yes	2293(95.94)	2237(95.93)	56(96.55)
No	96(4.02)	94(4.03)	2(3.45)
N/A	1(0.04)	1(0.04)	0
Clubbing			
Yes	1(0.04)	1(0.04)	0
No	2388(99.92)	2330(99.91)	58(100)
N/A	1(0.04)	1(0.04)	0
Lymphadenopathy			
Yes	7(0.29)	7(0.30)	0
No	2382(99.67)	2324(99.66)	58(100)
N/A	1(0.04)	1(0.04)	0
Otitis media			
Right ear	3(0.13)	3(0.13)	0
Left ear	1(0.04)	1(0.04)	0
None	2373(99.29)	2316(99.31)	57(98.28)
N/A	13(0.54)	12(0.52)	1(1.72)
Stridor			
Yes	27(1.13)	27(1.16)	0
No	2361(98.79)	2303(98.76)	58(100)
N/A	2(0.08)	2(0.09)	0
Oral candida			
Yes	13(0.54)	11(0.47)	2(3.45)
No	2377(99.46)	2321(99.53)	56(96.55)
Oedema			
Generalised	1(0.04)	1(0.04)	0
None	2386(99.83)	2329(99.87)	57(98.28)
Pre-t	1(0.04)	0	1(1.72)
Pre-tib	2(0.08)	2(0.09)	0
Skin desquamation			
Yes	5(0.21)	5(0.21)	0

No	2385(99.79)	2327(99.79)	58(100)
Spleen enlargement (cm)			
0	2321(97.11)	2266(97.17)	55(94.83)
1	35(1.46)	33(1.42)	2(3.45)
2	18(0.75)	17(0.73)	1(1.72)
3	6(0.25)	6(0.26)	0
4	4(0.17)	4(0.17)	0
5	2(0.08)	2(0.09)	0
13	3(0.13)	3(0.13)	0
15	1(0.04)	1(0.04)	0
Vomiting			
Yes	435(18.20)	425(18.22)	10(17.24)
No	1955(81.80)	1907(81.76)	48(82.76)
Crackles			
Yes	1396(58.41)	1361(58.36)	35(60.34)
No	994(41.59)	971(41.64)	23(39.66)
Unable to drink			
Yes	233(9.75)	218(9.35)	15(25.86)
No	1606(67.20)	1575(67.54)	31(53.45)
N/A	551(23.05)	539(23.11)	12(20.69)
Head nodding			
Yes	416(17.41)	404(17.32)	12(20.69)
No	1425(59.62)	1391(59.65)	34(58.62)
N/A	549(22.97)	537(23.03)	12(20.69)
Breathing difficulty			
Yes	2243(93.85)	2188(93.83)	55(94.83)
No	146(6.11)	143(6.13)	3(5.17)
N/A	1(0.04)	1(0.04)	0
Bradycardia			
Yes	23(0.96)	22(0.94)	1(1.72)
No	2365(98.95)	2308(98.97)	57(98.28)
N/A	2(0.08)	2(0.09)	0

Supplementary table 1: Clinical characteristics of the cohort.

**Characteristic**	**Overall (N=2390)**	**Survivors (n=2332)**	**Deaths (n=58)**
Hemoglobin, g/ dL, median (IQR)	9.7 (8.9-10. 5)	9.7 (8.9-10.5)	8.75 (7.83-10.05)
RBC count, $\times 10^6/\mu L$, median (IQR)	4.15 (3.67-4.59)	4.16 (3.68-4.60)	3.91 (3.11-4.23)
WBC count, $\times 10^3/\mu L$, median (IQR)	12 (9.1-16.1)	12 (9.1-16.0)	15.25 (9.90-18.85)

Platelets, $\times 10^3/\mu L$, median (IQR)	467 (370-574.25)	469.5 (373-576.75)	357 (234.50-465.75)
Mean Cell Volume (MCV) (fL), median (IQR)	73.1 (65.63-82)	73.05 (65.70-82)	76.05 (62.33-83.00)

Supplementary table 2: Baseline haematological characteristics of the cohort

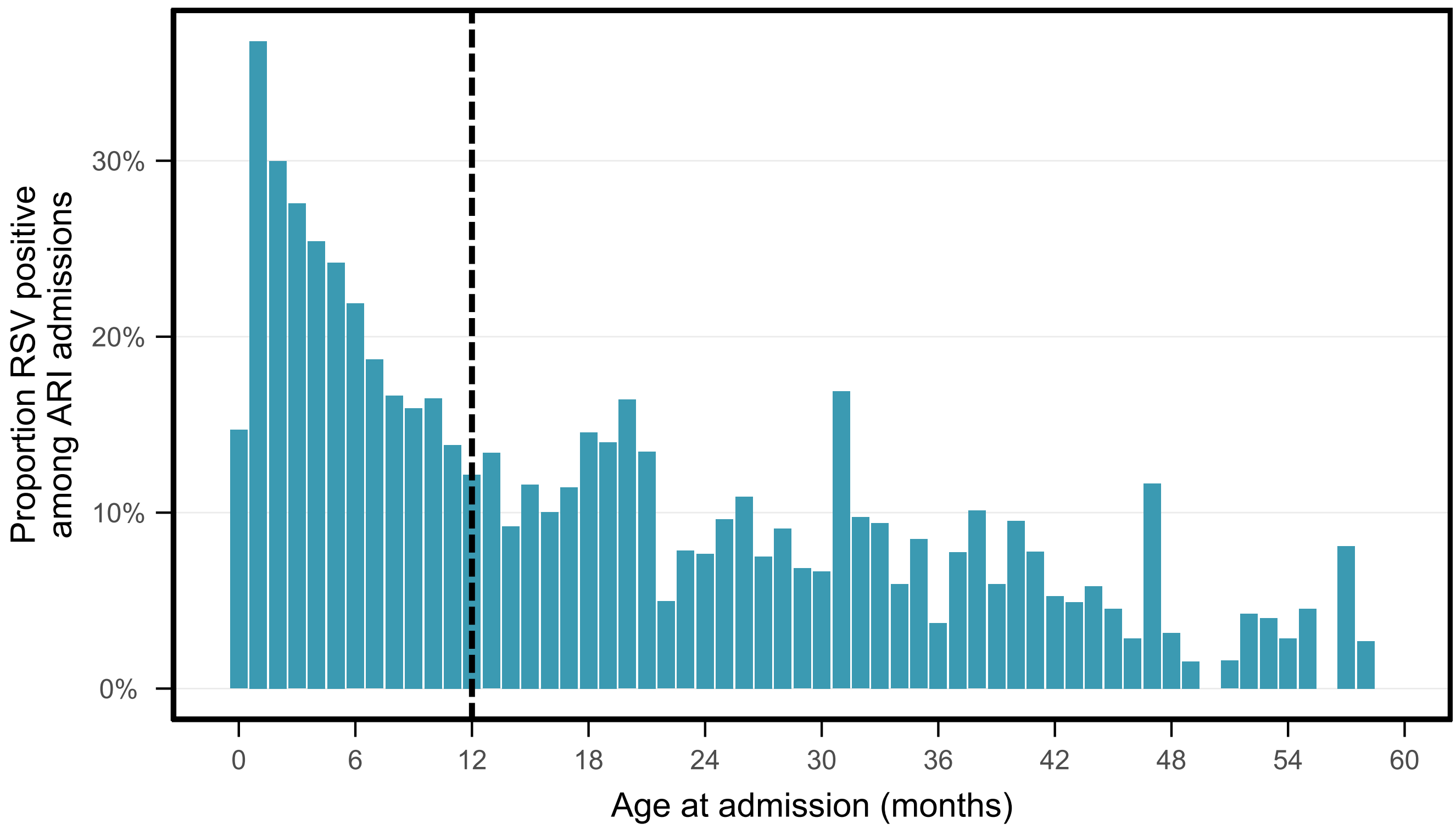

Supplementary Figure 1. Proportion of paediatric hospital admissions testing positive for respiratory syncytial virus (RSV) among children aged <5 years, 2001–2025. The figure shows the annual and age-specific proportion of hospital admissions with laboratory-confirmed RSV infection among children younger than five years admitted to Kilifi County Hospital. Admission prevalence was highest in the second month of life, during which approximately one-third of hospitalised infants tested positive for RSV.

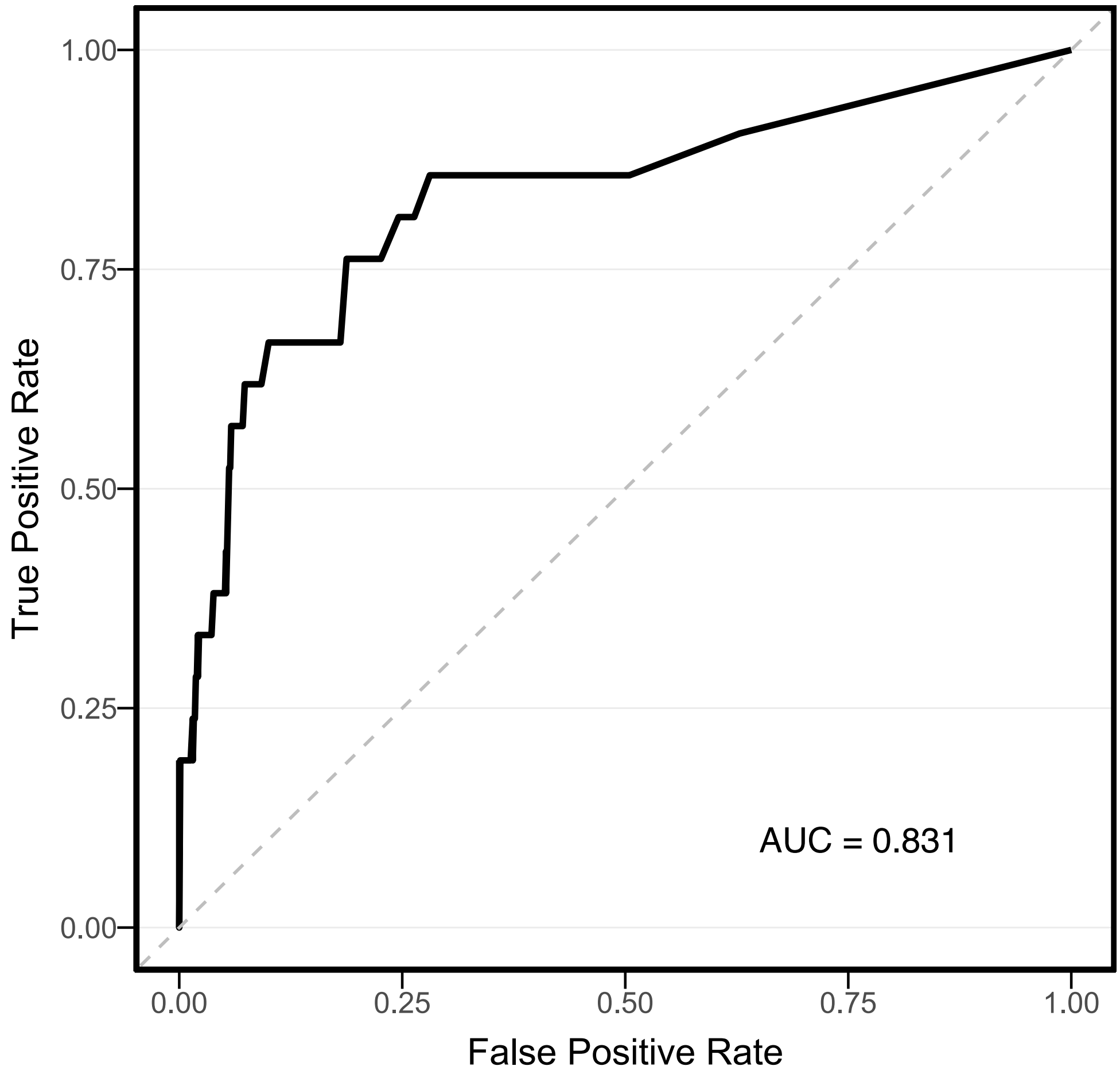

Supplementary Figure 2: Receiver Operating Characteristic (ROC) curve evaluating model performance. The x-axis indicates specificity, and the y-axis indicates the sensitivity. The model achieved an area under the receiver operating characteristic curve of 0.83.

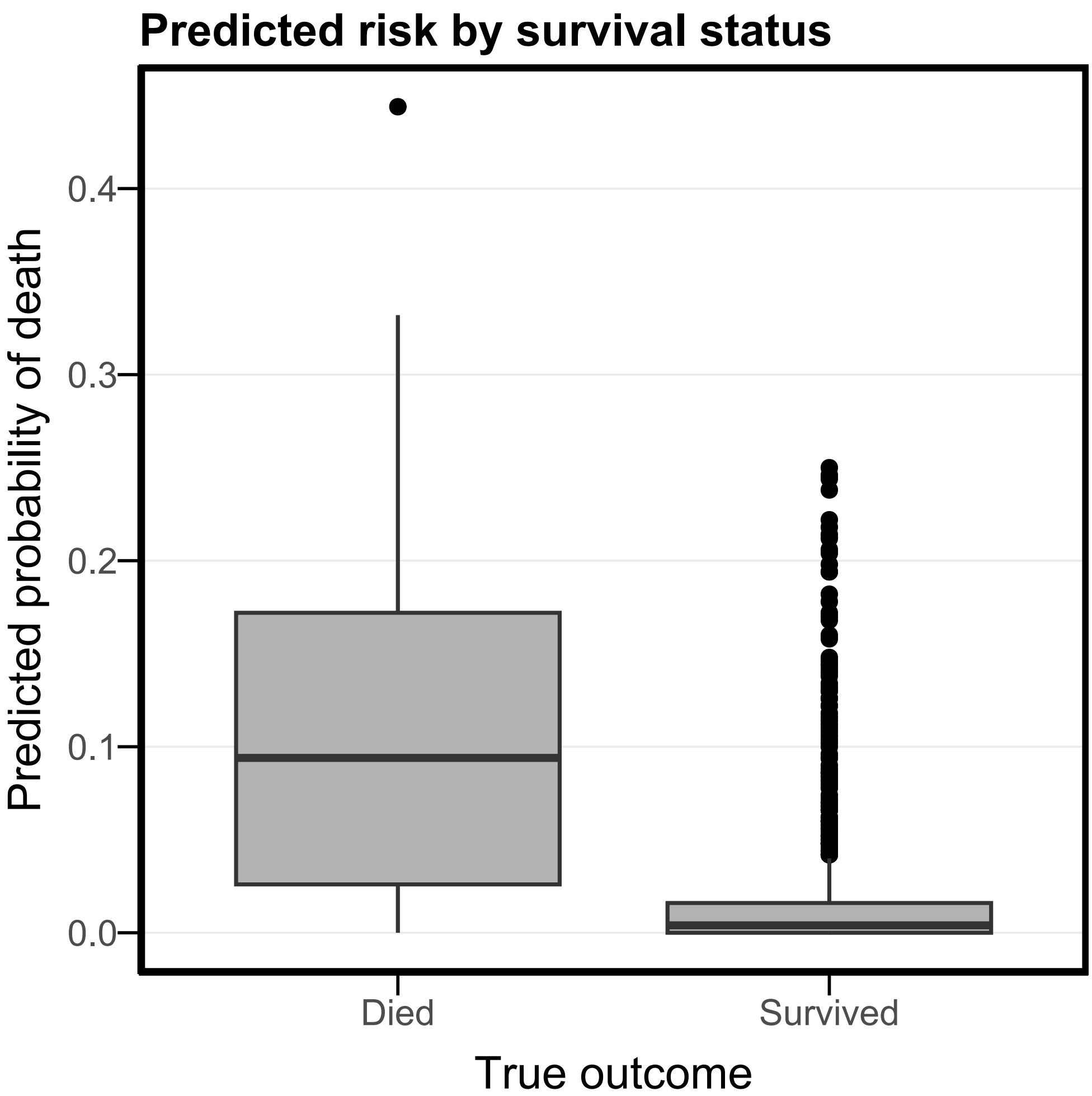

Supplementary Figure 3: Random forest–predicted probabilities of in-hospital death among RSV-positive infants, stratified by observed survival status. Infants who died (left) were assigned consistently higher predicted risks compared with those who survived.

Period	RSV positive (N)	HIV positive (N)	HIV prevalence (%)
2001-2005	268	0	0
2006-2010	632	15	2.37
2011-2015	660	22	3.33
2016-2020	473	10	2.11
2021-2025	357	0	0

Supplementary Table 3. HIV prevalence among RSV-positive infants by calendar period. Annual RSV-positive cases were grouped into 5-year periods. For each period, the total number of RSV-positive infants, the number who were HIV-positive, and the corresponding HIV prevalence (%) are shown.

Age (months)	died Median (IQR)	survived Median (IQR)
1	-3.50 (-3.90–-1.40)	-0.30 (-1.00–0.40)
2	-2.20 (-4.00–-0.30)	0.20 (-0.60–1.00)
3	-1.10 (-2.70–0.00)	0.10 (-0.60–0.80)
4	-1.30 (-1.80–-1.20)	-0.10 (-0.80–0.60)
5	-2.10 (-3.20–-0.80)	-0.40 (-1.20–0.20)
6	-3.20 (-4.10–-1.30)	-0.90 (-1.60–0.00)
7	-2.25 (-2.50–-2.00)	-1.20 (-2.00–-0.30)
8	-1.40 (-1.40–-1.40)	-1.30 (-2.00–-0.80)
9	-3.20 (-4.30–-2.90)	-1.50 (-2.10–-0.85)
10	-4.10 (-4.30–-2.00)	-1.90 (-2.70–-1.20)
11	-3.05 (-6.20–0.10)	-1.60 (-2.70–-0.90)
12	-2.60 (-3.10–-2.20)	-2.00 (-2.40–-1.50)

Supplementary table 4: weight-for-age z-scores for RSV survivors and non-survivors

Age (months)	died Median (IQR)	survived Median (IQR)
1	7.60 (7.00–10.00)	11.20 (10.40–12.00)
2	9.00 (7.00–10.40)	12.00 (11.00–13.00)
3	10.50 (9.00–11.90)	12.80 (11.80–13.50)
4	11.65 (11.00–12.20)	13.00 (12.10–14.00)
5	11.00 (9.30–12.50)	13.00 (12.00–14.00)
6	11.00 (9.00–11.50)	13.00 (12.10–14.00)
7	11.75 (11.50–12.00)	13.20 (12.00–14.30)
8	14.00 (14.00–14.00)	13.20 (12.40–14.00)
9	10.20 (8.00–11.50)	13.20 (12.55–14.00)
10	11.00 (10.00–13.10)	13.20 (12.30–14.20)
11	11.30 (10.60–12.00)	13.50 (12.70–14.50)
12	12.00 (10.20–14.00)	13.20 (12.80–14.00)

Supplementary table 5: Mid upper arm circumference (muac) for RSV survivors and non-survivors

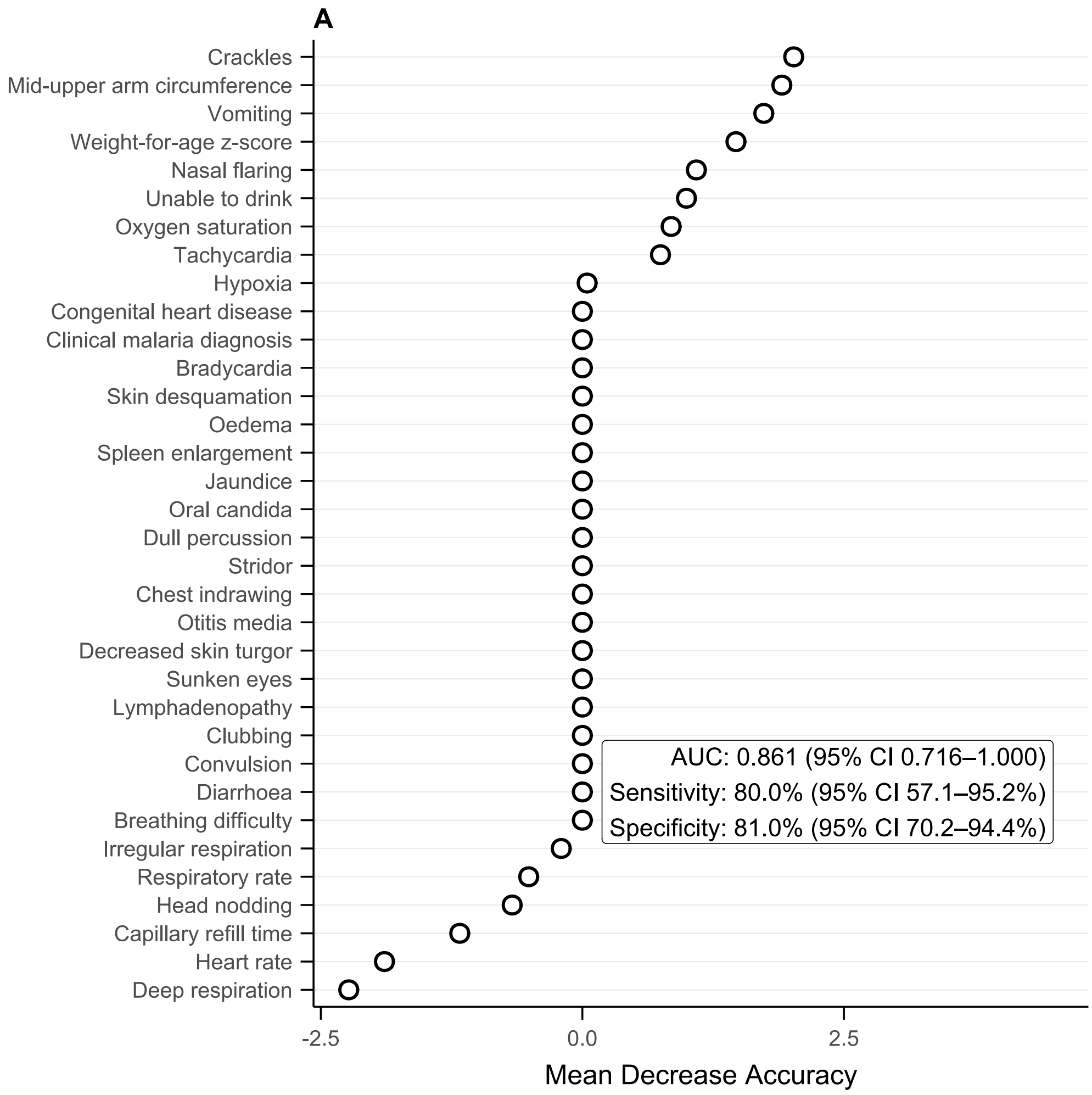

Supplementary figure 4: Random forest variable importance plot showing predictors of mortality among RSV-positive neonates. Clinical signs of respiratory distress and anthropometric vulnerability, including crackles, vomiting, lower weight-for-age z-score, and smaller mid-upper arm circumference, ranked highest in importance for mortality classification.

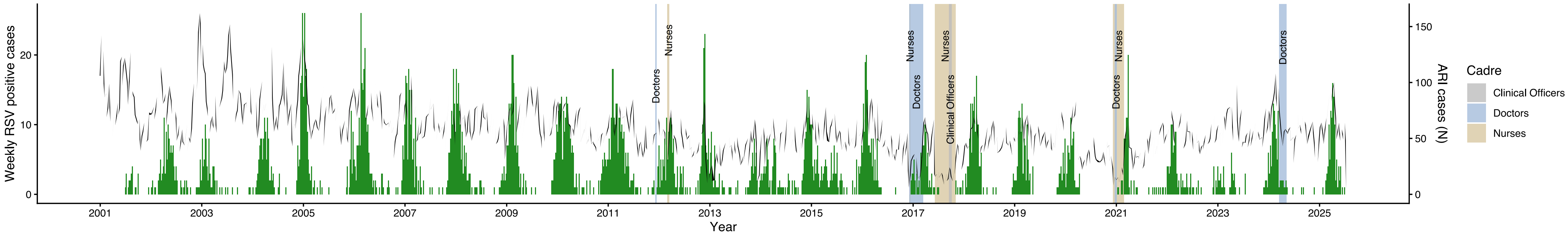

Supplementary figure 5. RSV epidemic waves and surveillance continuity over 25 years in Kilifi, Kenya. Weekly counts of RSV-positive cases (green bars) and number of children recruited into the study from 2001 to 2025 (black line). Shaded regions indicate periods of government healthcare worker strikes by cadre.

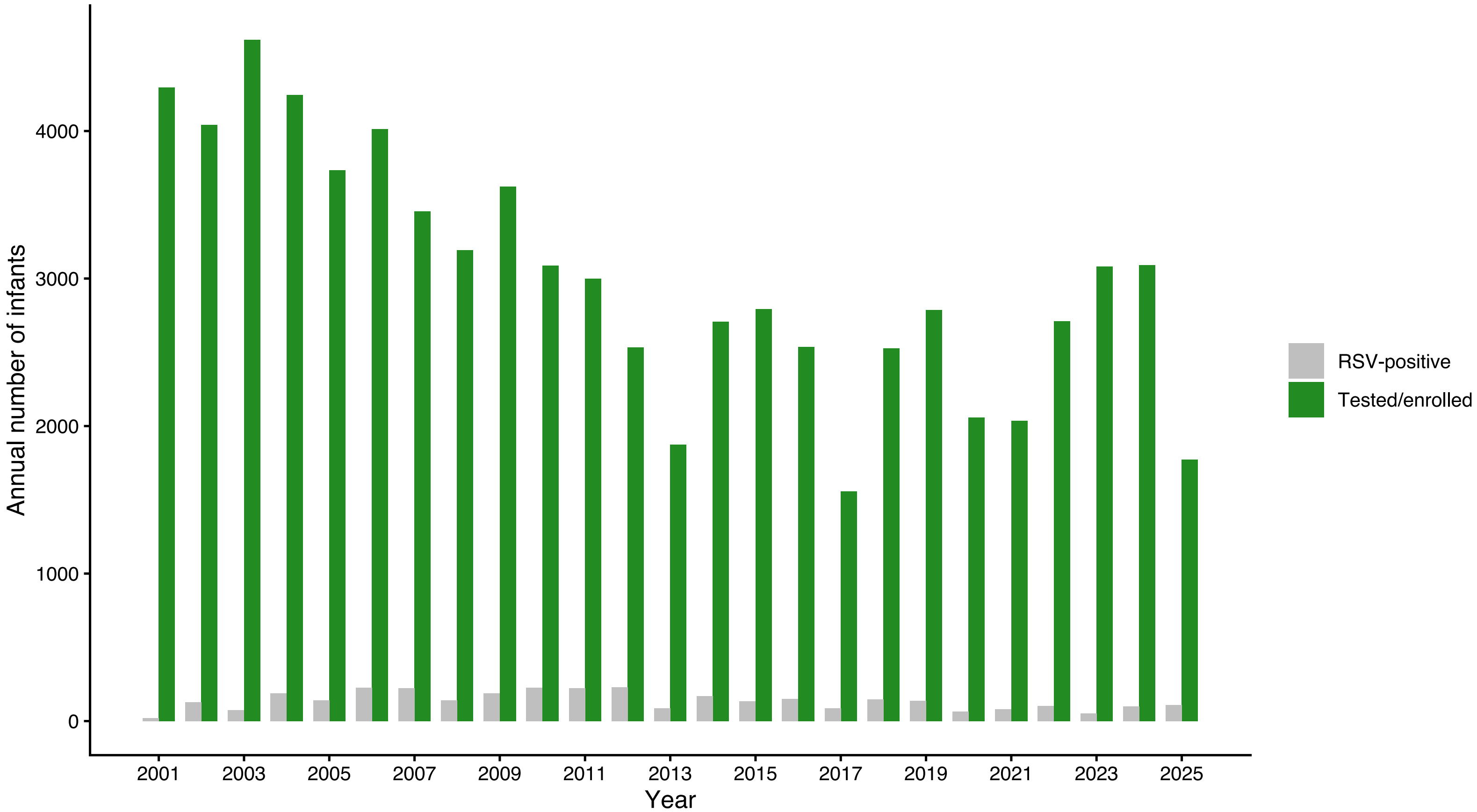

Supplementary figure 6. Annual enrolment and RSV-positive case counts over the 25-year surveillance period. Bars show the annual number of infants enrolled (green) and the number of RSV-positive cases by calendar year (gray)
